# Fusing MEMS technology with lab-on-chip: nanoliter-scale silicon microcavity arrays for digital DNA quantification and multiplex testing

**DOI:** 10.1038/s41378-020-00187-1

**Published:** 2020-10-05

**Authors:** Daniel Podbiel, Franz Laermer, Roland Zengerle, Jochen Hoffmann

**Affiliations:** 1 Robert Bosch GmbH, Corporate Sector Research, Microsystems and Nanotechnologies, Robert-Bosch-Campus 1, 71272 Renningen, Germany; 2 IMTEK - Department of Microsystems Engineering, University of Freiburg Georges-Koehler-Allee 103, 79110 Freiburg, Germany

**Keywords:** Nanoscience and technology, Microfluidics, Engineering, Nanoscale devices, Environmental, health and safety issues

## Abstract

We report on the development of a microfluidic multiplexing technology for highly parallelized sample analysis via quantitative polymerase chain reaction (PCR) in an array of 96 nanoliter-scale microcavities made from silicon. This PCR array technology features fully automatable aliquoting microfluidics, a robust sample compartmentalization up to temperatures of 95 °C, and an application-specific prestorage of reagents within the 25 nl microcavities. The here presented hybrid silicon–polymer microfluidic chip allows both a rapid thermal cycling of the liquid compartments and a real-time fluorescence read-out for a tracking of the individual amplification reactions taking place inside the microcavities. We demonstrate that the technology provides very low reagent carryover of prestored reagents < 6 × 10^−2^ and a cross talk rate < 1 × 10^−3^ per PCR cycle, which facilitate a multi-targeted sample analysis via geometric multiplexing. Furthermore, we apply this PCR array technology to introduce a novel digital PCR-based DNA quantification method: by taking the assay-specific amplification characteristics like the limit of detection into account, the method allows for an absolute gene target quantification by means of a statistical analysis of the amplification results.

## Amplifying the scale of DNA amplification

An integrated microfluidic device allows researchers to simultaneously detect and quantify multiple target DNA sequences in a single sample. A technique called the polymerase chain reaction (PCR) efficiently generates many copies of a given DNA sequence, even from tiny amounts of source material. Daniel Podbiel and colleagues at Robert Bosch GmbH in Germany have now developed a sophisticated device that can simultaneously perform many PCR reactions from one sample. Within their chip, the DNA is partitioned into multiple reaction wells, each of which can contain reagents needed to PCR amplify a different gene. These reactions are then performed on the chip, generating a fluorescence signal that can be detected and measured to reveal the amount of amplified DNA. Such a device could ultimately prove useful for conducting multi-gene analysis in medical diagnostics or other applications.

## Introduction

The amplification and detection of multiple DNA targets via polymerase chain reaction (PCR) has become a widely used technique in molecular diagnostic testing. Within the past two decades, several microfluidic devices have been developed for PCR-based sample analysis via geometric multiplexing in micro-^1–7^, nano-^8–21^, or even pico-^22–27^ liter sized reaction compartments by making use of microwell or microchamber arrays^8–15,22–25,28–30^, throughhole arrays^16,17^, evacuated compartments^1^, micro-capillaries^18^, a capillary-driven compartmentalization^2,19^, a centrifugal aliquoting^3–5,31,32^, the slipping of fluidic layers^11^, droplet arrays^20,33^, or droplet in oil systems^6,21,26,27,31,32,34,35^. The downsizing of reaction volumes and the development of novel microfluidic devices was driven by different motivations and application cases, for example, to enable a low-cost high-throughput testing^16,17,22^ or to allow for an absolute target DNA quantification by means of digital PCR^15,19,21,23,26,27,36–41^.

In view of these technological advances, we see great potential to empower molecular diagnostic testing at the point-of-care (PoC) with microfluidic multiplexing technologies. However, from a technological point of view, a PoC analysis calls for an automation of the entire workflow, including liquid aliquoting, thermal cycling, and (real-time) fluorescence read-out of the amplification reactions, as well as an implementation into a microfluidic LoC platform^42–46^. Hence, there remains an unmet need for the development of highly integrated microfluidic multiplexing technologies that are compatible with sample-to-answer PoC analysis systems. From a methodological point of view, the quantification of DNA by means of standard digital PCR is restricted to highly sensitive assays with a limit of detection (LOD) less than or equal to one DNA copy per reaction compartment. Regarding previous studies (see for example Fig. 4 in ref. ^8^, Fig. 2 in ref. ^9^, Fig. 4 in ref. ^11^, and Table 1 in ref. ^47^), a more generalized quantification methodology taking the assay-specific amplification characteristics into account appears indispensable for an accurate DNA quantification in such devices.

Here, we face these challenges by pursuing the strategy of fusing MEMS technology with lab-on-chip: the developed PCR array technology enables a fully automated aliquoting of a sample liquid inside an array of nanoliter-scale microcavities made from a silicon substrate and subsequent multiplex sample analysis via quantitative PCR (qPCR). The highly integrated hybrid silicon–polymer approach unites capillary-assisted aliquoting microfluidics, rapid thermal cycling, and real-time fluorescence read-out in a single microfluidic chip with a prestorage of dried reagents inside the microcavities, a robust liquid compartmentalization up to temperatures of 95 °C, and a spatially homogenous thermalization of all reaction compartments. We apply this PCR array technology to demonstrate both multiplex testing, as well as absolute DNA quantification thereby introducing a generalized methodology referred to as probability of detection (POD)-based digital PCR.

## Results and discussion

### Conceptualization of PCR array technology

**Fig. 1:**
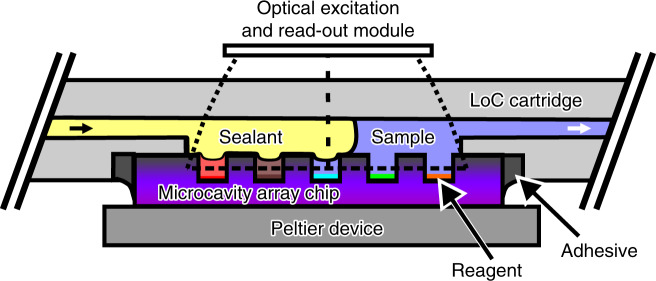
Concept of the PCR array technology. Aliquoting of a sample liquid in an array of microcavities; rapid thermal cycling using an external Peltier device; and real-time optical fluorescence read-out for a tracking of the individual quantitative PCR amplifications taking place inside the microcavities

 Figure 1 shows a sketch that illustrates the basic concept of the PCR array technology. A microcavity array chip made from a silicon substrate is implemented into a polymeric LoC cartridge in such a way that it features three kinds of interfaces: a microfluidic interface to fill the microcavities with a sample liquid via an inlet channel and to subsequently seal the microcavities with a second immiscible sealant liquid; a thermal interface for an exchange of heat with an external Peltier device enabling a rapid and homogeneous thermal cycling of the liquid aliquots inside the microcavities; and an optical interface for optical excitation and real-time fluorescence read-out of the liquid aliquots allowing for a tracking of the individual qPCR amplification reactions. Inside the microcavities, dried reagents like target-specific primers and probes or template DNA can be prestored. A flexible adhesive is used to bond the silicon chip fluidically tightly to the polymeric LoC cartridge for temperatures up to 95 °C.

In the following, we will proceed with an in-depth experimental characterization of this PCR array technology. Details regarding the fabrication technology of the microfluidic chips, the test setup used for carrying out the experiments, as well as the methodology employed for data acquisition and analysis are described in the “Materials and methods” section. The reader is recommended to read this section at first before continue reading the “Results and discussion” section.

For a characterization of the PCR array technology, we performed several experiments in order to investigate the cross talk between adjacent reaction compartments during thermal cycling, the carryover of prestored reagents during microfluidic filling and sealing, and the target-specific PCR amplification in selected microcavities by means of prestored primers and TaqMan probes. Besides the characterization of the technology, we describe a novel method for the absolute quantification of DNA in a sample liquid. The method enhances the quantification capabilites of standard digital PCR by incorporating the detailed launch characteristics of an amplification reaction inside a compartment under defined environmental conditions. By applying this probability of detection (POD)-based digital PCR, an accurate DNA quantification can be accomplished in multi-compartment devices by making use of amplification reactions, with arbitrary sensitivity characteristics and a LOD greater than one.

### Absolute DNA quantification by POD-based digital PCR

**Fig. 2:**
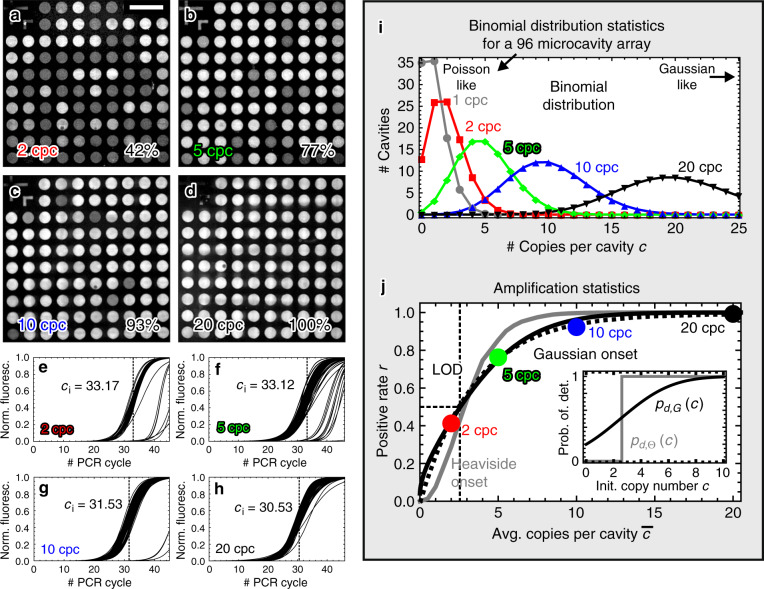
Amplification statistics for the detection of the ABL gene target within the microcavity array for four different copy numbers. **a**–**d** Endpoint fluorescence micrographs acquired at the annealing temperature (60 °C) corresponding to 2, 5, 10, and 20 initial calculated copies per microcavity. The scalebar in **a** corresponds to 1 mm. The micrographs are depicted in a contrast adjusted grayscale representation. **e**–**h** Corresponding normalized fitted amplification curves. The average *c*_*i*_ values are indicated by the vertical dashed lines and are numerically displayed in the graphs. **i** Binomial distribution statistics for a 96 microcavity array for initial average copy numbers $\bar{c}$ of 1, 2, 5, 10, and 20 copies per cavity as indicated in the graph. **j** Relation between the initial average copy number $\bar{c}$ and the experimentally measured positive rate *r*. By making use of a suitable probability of detection-based launch function *p*_*d*_(*c*) the experimental data can be well reproduced by the model

For an experimental investigation of the PCR performance within the microcavity array chip, we introduced a liquid PCR master mix into the microcavities that contained primers and probes for the detection of the ABL (Abelson murine leukemia viral oncogene homolog 1, also known as ABL1) gene target associated with chronic myeloid leukemia (CML), as well as corresponding template DNA (see “Materials and methods” section and ref. ^48^ for further information). In this case, no PCR reagents were prestored inside the microcavities. However, different concentrations of ABL template DNA within the liquid master mix were used. The obtained test results are summarized in Fig. 2a–d showing four endpoint fluorescence micrographs acquired after thermal cycling at the annealing temperature (60 °C), which correspond to initial template DNA concentrations of $\bar{c}=$ 2, 5, 10, and 20 calculated copies “per” microcavity (cpc) on average; Fig. 2e–h shows the corresponding normalized fitted amplification curves (see “Materials and methods” section for data analysis methodology).

The endpoint fluorescence micrographs in Fig. 2a–c indicate the presence of different fluorescence signal levels inside the individual microcavities after thermal cycling. In particular, an amplification-related sigmoidal increase in the fluorescence signal is detected in a certain fraction of the microcavities only. The fraction varies from 42% for 2 cpc, over 77% for 5 cpc, and 93% for 10 cpc, up to 100% for 20 cpc (see bottom right corners of Fig. 2a–d). Hence, the measurements provide clear evidence that the portion of microcavities where a PCR takes place during thermal cycling is related to the initial average copy number per microcavity $\bar{c}$.

Since all experiments were conducted under the same environmental conditions, the observed variation of the positive rate must be ascribed to the specific amplification characteristics like the sensitivity and the selectivity of the used PCR. For a general quantitative analysis of the amplification results, the binomial distribution statistics ${B}_{n}(c,\bar{c})$ have to be taken into account, which describe the randomly generated initial copy number distribution inside the microcavity array. That is, ${B}_{n}(c,\bar{c})$ gives the probability for the case that a compartment comprises exactly *c* copies, if the average copy number in each of the *n* compartments is given by $\bar{c}$. Figure 2i illustrates the distribution statistics for a *n* = 96 compartmentalization, with $\bar{c}=$ 1, 2, 5, 10, or 20 cpc on average as given by the binomial probability density function

1 $${B}_{n}(c,\bar{c})=\left(\begin{array}{l}n\cdot \bar{c}\\ c\end{array}\right){n}^{-c}{(1-1/n)}^{n\cdot \bar{c}-c}$$

${B}_{n}(c,\bar{c})\cdot n$ describes the number of microcavities comprising exactly *c* initial copies of DNA, which is plotted there. As indicated in Fig. 2i, the general binomial statistical description includes both Poisson and Gaussian distribution statistics as limiting cases. In contrast to the Poisson distribution, which is commonly used in digital PCR analysis, the more general binomial distribution is also valid for the case that the number of reaction compartments *n* is rather small. However, for a relatively large number or reaction compartments *n*, the Poisson distribution $P(c,\bar{c})={\bar{c}}^{c}/c!\ {e}^{-\bar{c}}$ serves a good approximation, i.e., ${B}_{n}(c,\bar{c})\to P(c,\bar{c})$ for *n* → *∞*, and might be used as well for calculation.

In order to relate the experimentally determined positive rates with the initial copy number distribution, it appears useful to introduce a characteristical POD function *p*_*d*_(*c*) that describes the probability for a launch of an amplification reaction inside a thermalized microcavity compartment (under defined environmental conditions) in dependence on the initial number of DNA copies *c* inside the compartment. By applying the binomial distribution statistics ${B}_{n}(c,\bar{c})$, the fraction *r* of compartments where an amplification reaction takes place may be described by a POD-based binomially estimated launch rate

2 $$r(\bar{c})=\mathop{\sum }\limits_{c=0}^{\infty }{p}_{d}(c)\cdot {B}_{n}(c,\bar{c})$$

Besides the discrete formulation using sums an integral description can be employed as well as given in the Supplementary Information.

The POD function *p*_*d*_(*c*) may be approximated by a Heaviside function Θ with a step at the copy number *c* = *c*_LOD_ corresponding to the LOD of the amplification reaction:

3 $${p}_{d,\Theta ;{\rm{LOD}}}(c)=\Theta (c-{c}_{{\rm{LOD}}})$$

In this simple approximation, the ratio *r* of reaction compartments where an amplification takes place, as given in Eq. (2), becomes

4 $${r}_{\Theta ;{\rm{LOD}}}(\bar{c})=\mathop{\sum }\limits_{c={c}_{{\rm{LOD}}}}^{\infty }\ {B}_{n}(c,\bar{c})$$

For a more realistic description of the onset of an amplification reaction inside a compartment, it appears viable to include a detection uncertainty to the POD function by folding the Heaviside function *p*_*d*,Θ;LOD_(*c*) with a Gaussian probability density function of width *w*

5 $${G}_{w,{c}_{0}}(c)=1/\sqrt{2\pi {w}^{2}}\cdot \exp [-{(c-{c}_{0})}^{2}/(2{w}^{2})]$$

in order to incorporate a continuous onset of the amplification reaction around the LOD copy number *c*_LOD_ into the POD function

6 $${p}_{d,G;{\rm{LOD}},w}(c)={\int\nolimits_{\!-\infty }^{\infty }}dc^{\prime} \ {p}_{d,\Theta ;{\rm{LOD}}}(c-c^{\prime} )\ {G}_{w,{c}_{0} = 0}(c^{\prime} )$$

yielding a POD-based binomially estimated launch rate of

7 $${r}_{G;{\rm{LOD}},w}(\bar{c})=\mathop{\sum }\limits_{c=0}^{\infty }\left({\int\nolimits_{\!-\infty }^{c-{c}_{{\rm{LOD}}}}}dc^{\prime} \ {G}_{w,0}(c^{\prime} )\right)\cdot {B}_{n}(c,\bar{c})$$

The graph in Fig. 2j compares the experimental results with calculative results from the analytical model. The experimentally measured positive rates *r* are plotted as labeled colored dots in dependence on the calculated initial average copy number per microcavity $\bar{c}$. The POD-based binomially estimated launch rates $r(\bar{c})$ from the model described above are indicated by the gray and black line, representing the Heaviside and the Gaussian onset modeling, respectively. The fitting parameters are *c*_LOD_ = 2.6 for the Heaviside onset model *p*_*d*,Θ_(*c*) and *c*_LOD_ = 2.5, *w* = 2.95 for the Gaussian onset model *p*_*d*,*G*_(*c*). The inset in Fig. 2j indicates the two corresponding POD functions *p*_*d*,Θ;LOD_(*c*) and *p*_*d*,*G*;LOD,*w*_(*c*).

Both POD functions reproduce the increase in the positive rate *r* that is associated with the average copy number $\bar{c}$ inside the compartments. However, the Gaussian onset model with a continous probability variation shows a better quantitative agreement with the experimental data. The coefficients of determination *R*^2^ yield ${R}_{\Theta }^{2}=88.19 \%$ for the Heaviside onset model and ${R}_{G}^{2}=99.20 \%$ for the Gaussian onset model, respectively. By applying the Gaussian onset model, a 50% LOD of about *c*_LOD_ = 2.5 (see above) can be infered from the experimental data. The measured *c*_*i*_ values given in Fig. 2e–h are consistent with the results and indicate an efficient amplification reaction. All in all, the model and the experimental results are in good agreement.

Notably, the positive rates obtained from the Gaussian onset model are similar to a heuristic approach based on the Poissonian fraction $1-{e}^{-\bar{c}}$ of compartments comprising at least one copy. By introducing a LOD copy number *c*_LOD_ into the calculation of this fraction, one obtains a launch rate of ${r}_{P;{\rm{LOD}}}(\bar{c})=1-{e}^{-\bar{c}/{c}_{{\rm{LOD}}}}$. The corresponding fit with *c*_LOD_ = 3.5 is indicated by the dashed black line in Fig. 2j, and shows good agreement with both the experimental positive rates, as well as the Gaussian onset modeling approach. Accordingly, further experimental studies should investigate if an onset modeling based on a given LOD copy number might be sufficient for an accurate digital DNA quantification, or if a more general POD function-based approach is advantageous in particular cases.

### Estimation of the cross talk rate during thermal cycling

An important property of every compartment-based reaction device is the cross talk rate between adjacent reaction compartments. For our case, the cross talk rate may be defined as the fraction of PCR-generated amplification product in a microcavity that is accidentally transferred to another (adjacent) microcavity during one PCR cycle.

In general, cross talk might be a serious issue that crucially affects the occurence of false positives and the entire functionality of the reaction device. However, the experiments presented in Fig. 2 demonstrate that the cross talk rate of our microcavity arrays must be very low in general, in the order of only 10^−3^ per PCR cycle:

A significant cross talk between adjacent microcavities in the order of 10^−1^ per PCR cycle would lead to a high false-positive rate with the consequence, that all microcavities would generate a positive fluorescence signal within a certain amount of thermal cycles. The micrographs in Fig. 2a–c and the observed amplification statistics proof that this is not the case here. However, a close look onto the amplification curves in Fig. 2e–h reveals that there are bunches of amplification curves corresponding to an average *c*_*i*_ value that is about ten cycles higher than the main amplification peak. The large shift of about ten PCR cycles cannot be explained by the (small) variation of the initial copy number inside the microcavities due to the binomial distribution statistics: even an initial copy number variation by a factor of 16 = 2^4^ (see Fig. 2i) would lead to a shift of about four PCR cycles only. Hence, the delayed amplification signals have to be false positives that may be ascribed to a possible cross talk between adjacent microcavities. However, the large delay of about ten PCR cycles indicates a maximum cross talk rate in the order of only 1/2^10^ = 1/1024 ≈ 10^−3^ per PCR cycle. Although the presence of a possible spatially confined cross talk between individual microcavities cannot be excluded by our methodology, the results proof a very low cross talk in general, which is acceptable for PCR-based testing.

### Determination of reagent carryover during microfluidic filling and sealing by the prestorage of template DNA in selected microcavities

In section “Absolute DNA quantification by POD-based digital PCR”, we derived from the amplification statistics a 50% LOD for the ABL gene target of ~2.5 cpc. Thus, due to the good sensitivity, the used PCR appears well suited for a characterization of a possible carryover of prestored reagents that may take place during filling and sealing of the microcavity array. For this purpose, we used a microcavity array where every second microcavity was loaded with 100 copies of ABL template DNA. The master mix, which was introduced into the flow chamber, contained no template DNA but the corresponding primers and probes for the amplification and detection of the ABL gene target.

**Fig. 3:**
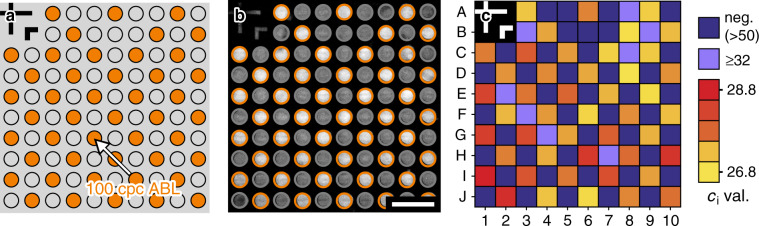
Experimental investigation of reagent carryover during microfluidic filling and sealing of the microcavity array by PCR-based amplification of prestored ABL template DNA. **a** Chessboard-like spotting layout. Every second microcavity contains some 100 copies of ABL template DNA. **b** Fluorescence micrograph acquired during thermal cycling at annealing temperature (60 °C). The scalebar corresponds to 1 mm. **c** False color map of the *c*_*i*_ values of the polymerase chain reactions inside the individual microcavities. The microcavities where no PCR amplification could be detected within 50 temperature cycles are indicated in dark blue

 Figure 3a sketches the chessboard-like spotting layout. Regarding a possible carryover of prestored reagents, this pattern represents the most critical scenario, where a carryover of only a few copies of DNA from one of four adjacent microcavities into another will cause a false-positive amplification signal in that microcavity (in the Supplementary Information another spotting layout is investigated in addition).

 Figure 3b shows a fluorescence micrograph that was captured during thermal cycling at the annealing temperature (60 °C). The image illustrates that a positive PCR amplification signal is generated in every microcavity containing prestored template DNA (as marked by the orange circles). In contrast, the microcavities that does not contain template DNA at the beginning (marked by the gray circles) show an overall weaker fluorescence signal. Hence, considering the very low cross talk rate of our microcavity array (see last section), this observation indicates that also no significant carryover of prestored template DNA takes place during filling and sealing of the microcavity array chip.

However, to further investigate this issue, we analyzed the individual amplification curves in addition. Figure 3c shows a map of the measured *c*_*i*_ values of the amplification reactions, that is the temperature cycle numbers that correspond to the inflection points of the fitted amplification curves. The used false color-scale representation is given on the right side of Fig. 3c. Evidently, the *c*_*i*_ value false color map resembles a chessboard pattern. The microcavities with prestored template DNA inside show *c*_*i*_ values in the range between 26.8 and 28.8 (colored in yellow, orange, and red), while the microcavities without prestored template DNA show predominantly no amplification within 50 cycles of thermal cycling (colored in dark blue), or a delayed amplification signal (*c*_*i*_ ≥ 32, colored in light blue). All in all, the difference of the *c*_*i*_ cycle values between adjacent true-positive and false-positive amplification reactions is at least about four. Hence, based on the difference of the *c*_*i*_ cycle values corresponding to true-positive and false-positive microcavities, the maximum fraction of carried over template DNA can be estimated to some 1/2^4^ ≈ 6%. In the predominant areas, where no false-positive amplification signal is generated the fraction is even smaller. Based on the LOD of the used amplification reaction of about *c*_LOD_ = 2.5 determined in the previous section, the fraction can be estimated to a maximum of only some *c*_LOD_/100 = 2.5% in these areas.

In summary, the experiment demonstrated that the carryover of prestored reagents that takes place during filling and sealing of the microcavities is low in general. For multiplexing purposes with prestored target-specific primers and probes (but no DNA), the measured carryover rate should be fairly acceptable: even a carryover of some 10% would lead to concentrations of accidentally transferred primers and probes that are about ten times lower than the standard concentrations in an ordinary PCR master mix. Consequently, the primer and probe concentrations in such a microcavity were both far too low to facilitate an efficient PCR reaction and to generate a false-positive amplification signal. However, in the following section, we will address this issue experimentally.

### Multi-targeted sample analysis by the prestorage of specific primers and probes in selected microcavities

In the last two subsections, we demonstrated that the used microcavity chips enable a geometric multiplexing by means of qPCR with very low cross talk during thermal cycling, and minor reagent carryover during microfluidic filling and sealing. In the following, we will briefly address the possibility of a multi-targeted sample analysis by a prestorage of target-specific primers and probes inside single microcavities.

For a basic testing, we prestored two sets of primers and probes inside 12 specific microcavities each addressing the ABL or the e13a2 gene target, respectively. Both genes are associated with CML (ref. ^48^). For the ABL gene target, we used a Cy3 fluorescence TaqMan probe while for the e13a2 gene target a Cy5 TaqMan probe was used. The 12 microcavities used for each gene target were distributed in a hexagonal pattern across the entire microcavity array in such a way that all adjacent microcavities of a loaded microcavity are empty. Accordingly, a possible carryover of reagents to an adjacent microcavity or a cross talk between adjacent microcavities during thermal cycling should be clearly observable.

**Fig. 4:**
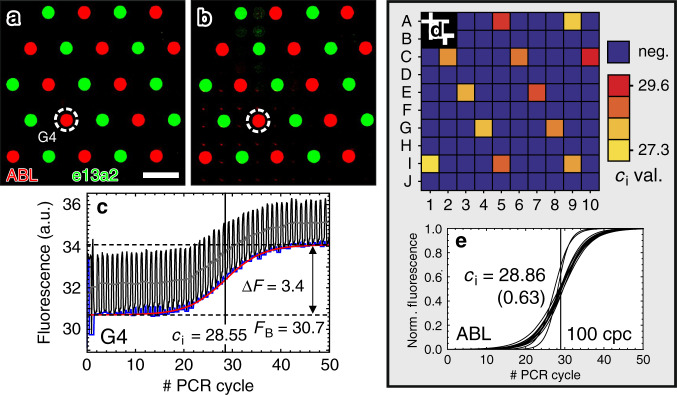
Detection of the ABL gene target in a sample liquid using specific prestored primers and probes. **a** Two-channel fluorescence micrograph taken before thermal cycling. The scalebar corresponds to 1 mm. **b** Endpoint two-channel fluorescence micrograph acquired after 50 PCR cycles. The micrographs in **a** and **b** are shown in the same false color representation in order to ensure visual comparability. **c** Fluorescence signal of microcavity “G4” during thermal cycling. **d** Map of the *c*_*i*_ values. **e** Normalized fitted amplification curves of the 12 microcavities with prestored primers and probes for the detection of the ABL gene target

Figure 4a, b shows two two-channel fluorescence micrographs in a RGB false color-scale representation before and after thermal cycling, respectively. The greyscale signal of the Cy3 fluorescence channel related to the ABL gene TaqMan probe is shown in red, while the Cy5 fluorescence signal of the e13a2 gene TaqMan probe is colored in green. Both micrographs are in good agreement, which indicates that there is no significant cross-contamination of adjacent microcavities due to carryover or cross talk. However, the 12 microcavities corresponding to the ABL gene target exhibited an increase in the fluorescence signal during thermal cycling.

The graph in Fig. 4c depicts a typical amplification-related fluorescence curve that corresponds to the microcavity “G4”. Like before, a sigmoidal increase of the fluorescence signal is observed. The *c*_*i*_ = 28.55 value is shifted to a lower value due to the higher concentration of ABL template DNA inside the master mix ($\bar{c}=100\ {\rm{cpc}}$).

 Figure 4d shows a map of the *c*_*i*_ value distribution inside the microcavity array, Fig. 4e a graph of the normalized fitted amplification curves. Evidently, an amplification is achieved in all microcavities with prestored ABL primers and probes while no false-positive amplification signals are generated. Hence, the microcavity chips used here appear well suited for multiplexing applications, where different sets of primers and probes are prestored in selected microcavities.

### Conclusions and outlook

Our experiments demonstrated that functionalized silicon-based microcavity array chips are an excellent component for PCR-based sample analysis in polymeric LoC cartridges. By a prestorage of specific primers and probes inside individual microcavities, multiple targets may be addressed within a single chip. Due to the established and highly developed silicon micromachining techniques, a further reduction of the reaction volumes and an increase in the degree of multiplexing seems possible. Hence, in this article, we introduced the concept of fusing MEMS technology with lab-on-chip. From our point of view, the used hybrid silicon–polymer approach is a key to combine the best of both worlds: a microcavity array chip made from silicon, on the one hand, featuring a metal-like heat conductivity for a rapid and spatially homogeneous thermal cycling of the reaction compartments, tailored wetting properties for a capillary-assisted, fully automatable, and temperature-stable microfluidic aliquoting of the sample liquid, a high fabrication accuracy to provide reaction compartments with a precisely defined volume, no significant self-fluorescence for precise fluorometric qPCR measurements, and an inert surface that facilitates miniaturized biochemical reactions. While a polymeric lab-on-chip cartridge, on the other hand, can provide active fluid management for pumping and valving of liquids, reservoirs for an on-chip long-term storage of reagents, a world-to-chip interface for an introduction of the sample, as well as an enclosure of the liquids for a safe and contamination-free sample processing. Following this approach, we will perform further tests inside a specifically designed Vivalytic LoC cartridge that enables a fully automated filling and sealing of the microcavity array in an external processing unit (see ref. ^49^). In this way, the filling and sealing dynamics become highly reproducible making an even more detailed investigation of reagent carryover possible. Within that context, different additives apart from polyethylene glycol (PEG) may be tested to further reduce the reagent carryover during microfluidic filling and sealing.

Furthermore, we described a digital DNA quantification method taking the assay-specific amplification characteristics like the LOD into account. It is likely that the description introduced here coincides well with the experimental results reported in previous studies (see, for example, Fig. 4 in ref. ^8^, Fig. 2 in ref. ^9^, Fig. 4 in ref. ^11^, and Table 1 in ref. ^47^). However, further studies should investigate if the introduced amplification onset modeling by a POD function *p*_*d*_(*c*) is a generally valid approach for a description of the assay-specific amplification characteristics, thus enabling an accurate quantification of DNA using different amplification reactions and compartment-based digital PCR devices.

In conclusion, by providing a highly parallelized and quantitative testing of a plurality of different gene targets, the here presented PCR array technology constitutes the basis for the implementation of complex bioassays into lab-on-chip systems with a broad range of possible applications in the PoC molecular laboratory diagnostics field.

## Materials and methods

### Wafer-level microcavity array fabrication and reagent dispensing into the microcavities

**Fig. 5:**
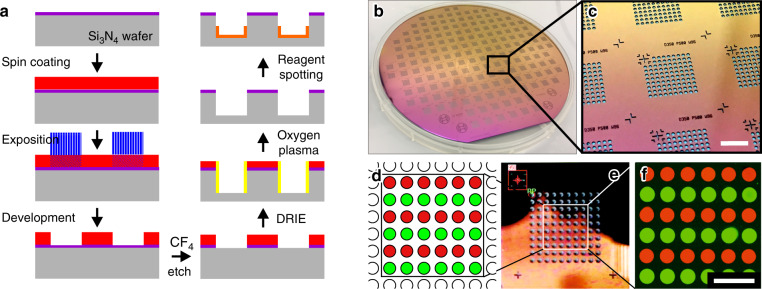
Silicon microcavity array chip fabrication and reagent spotting into the microcavities. **a** Sketch of the fabrication process route. The microcavities are trenched into the silicon wafer using DRIE. **b** Micromachined 6'' silicon wafer with 172 microcavity arrays before dicing. **c** Close-up view. Each array comprises 96 microcavities with a volume of 25 nl each. The scalebar corresponds to 3 mm. **d** Reagent spotting test layout with two different fluorescent dyes. **e** Automated reagent spotting into the microcavities is accomplished via optical detection of the reference mark in the top left corner of the chip. **f** Two-channel fluoresence micrograph (in a false color-scale representation) of the microcavity array chip after filling and sealing in a flow cell. The scalebar corresponds to 1 mm

For the fabrication of the microcavity array chips (see Fig. 5a), 6'' silicon wafers with a standard thickness of 675 μm were used as a substrate. After low-pressure chemical vapor deposition (centrotherm international AG) of 50 nm silicon oxide (SiO_2_) and 140 nm silicon nitride (Si_3_N_4_), the wafers were spin-coated (LabSpin6, Suess Microtec SE) at 4000 r.p.m. with a polymeric photoresist (AZ4562, Clariant) of 5–6 μm thickness. Subsequent to a soft bake on a hotplate (5 min at 107 °C), the photoresist was exposed in a mask aligner (MA150, Suess Microtec SE) using a photomask (digiraster GmbH, Co. KG, plotted with EIE-type RP212-NT) and finally developed (AZ 826 MIF, Clariant). Then, a CF_4_ plasma etching process (Multiplex ICP, Surface Technology System Ltd) was applied in order to remove the SiO_2_/Si_3_N_4_ coating from the areas not covered by the photoresist. Afterward, the microcavities were trenched inside the silicon substrate using deep reactive ion etching (DRIE, Multiplex ICP, Surface Technology System Ltd). In this way, the fabricated microcavities feature a nearly cylindrical shape with a diameter of 350 μm, a depth of 250 μm, and a volume of 25 nl. After DRIE, the remaining photoresist was removed by an oxygen plasma treatment. Finally, the wafers were diced by a wafer saw (DAD 320, Disco Hi-Tec Europe GmbH) to obtain chips with dimensions of 9 mm × 9 mm.

 Figure 5b shows a micromachined 6'' silicon wafer with 172 microcavity arrays before dicing. A close-up shot is depicted in Fig. 5c. After dicing, each chip features a quadratic array of 96 microcavities with a pitch (center–center) of 500 μm and an alignment mark in the top left corner of the array.

The reagents were introduced into the microcavities using an array spotting system (sciFLEXARRAYER S1, Scienion AG). The system employed a piezo dispensing capillary for the generation of microdroplets with a volume of ~300 pl enabling an accurate reagent deposition. In between two spotting steps, the piezo dispensing capillary was adequately washed with filtered and degassed deionized water to prevent cross-contamination of different reagents. The alignment mark in the top left corner of the microcavity array was optically detected by the system to serve as a reference point for a fully automated and reliable dispensing into the microcavities. In this way, the microcavities could be loaded with primers, probes or template DNA. In order to improve the wettability of the microcavities, a small amount of PEG (PEG 6000 or PEG 2000, Carl Roth GmbH, Co. KG) was added by array spotting finally.

 Figure 5d sketches a spotting test layout using two different fluorescent dyes colored in red and green, respectively. Figure 5e shows a micrograph of the array spotting systems’ camera. The recognized reference mark in the top left corner of the chip, and the deviated position of the top left microcavity of the array are highlighted by a red rectangle and a red circle, respectively. Figure 5f shows a two-channel fluorescence micrograph (in a RGB false color scale) of the microcavity array inside a microfluidic flow chamber after a resolution of the dried fluorescent dyes with an aqueous solution and a subsequent sealing of the microcavities with an immiscible sealant liquid.

### Hybrid silicon–polymer microfluidic chip fabrication

**Fig. 6:**
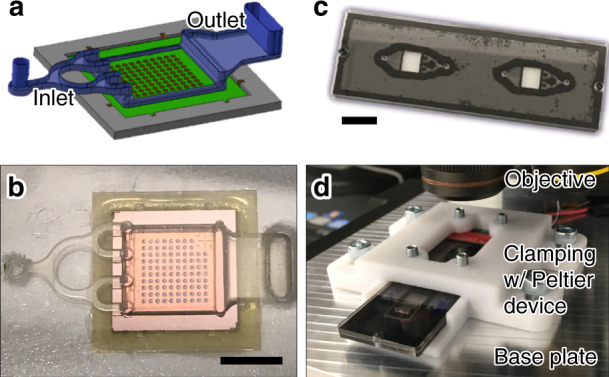
Silicon microcavity array chip implementation into a microfluidic polymer chip and experimental test setup. **a** Flow cell design with a branched inlet channel for a reliable microfluidic filling and sealing of the microcavity array chip. **b** Top-view micrograph of a flow cell made from a polycarbonate substrate. The silicon chip is joined with the substrate via adhesive bonding. The scalebar corresponds to 5mm. **c** Photograph of a laser-welded polymer chip comprising two flow cells for filling and sealing of two implemented silicon microcavity array chips. The scalebar corresponds to 10 mm. **d** Close-up view of the experimental setup used for thermal cycling and real-time fluorescence read-out of the microcavity array chips

**Fig. 7:**
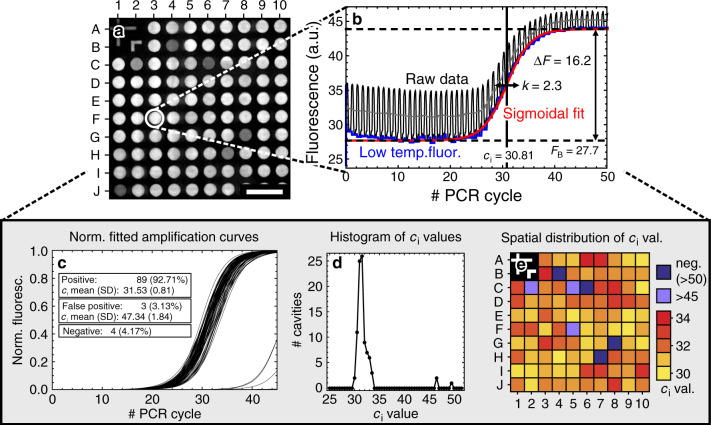
Data analysis of a PCR array measurement. **a** Fluorescence micrograph taken during the experiment. The scalebar corresponds to 1 mm. **b** Fluorescence signal from a single microcavity. The raw data are fitted by a sigmoidal curve. **c** Plot of the normalized fitted amplification curves from all 96 microcavities. **d** Histogram of the corresponding *c*_*i*_ values. **e** Spatial distribution of the *c*_*i*_ values

Regarding the implementation of the silicon microcavity array chip into a polymer-based microfluidic environment, we designed an appropriate flow cell that provides a reliable filling and sealing of all microcavities, by subsequently introducing the sample liquid and the sealant liquid via a common inlet port. Figure 6a shows a computer-aided design-based visualization of a microfluidic flow cell that was used to obtain the experimental results shown in Figs. 7 and  2. Figure 6b shows a corresponding top-view photograph of a manufactured polymeric test sample. In the particular design, the inlet channel branches symmetrically into four feeding channels that are connected via four throughholes to a flow chamber. The flow chamber has got a constant height of ~700 μm across the entire microcavity array. On the opposite side, the flow cell opens out into a broad outlet channel. For the experimental results depicted in Figs. 3 and 4, the same flow chamber was used but with a symmetrically shaped tapering of the inlet channel instead of a branching and one elongated throughhole instead of four separate throughhole connections.

For the fabrication of the test samples shown in Fig. 6b, c, we used injection-molded polycarbonate substrates with the dimensions of a microscope slide (75.5 mm × 25.5 mm × 1.5 mm), that were micromachined by an ultrashort pulse laser, namely a frequency-tripled neodymium yttrium-aluminum-garnet (Nd:YAG) laser (Lumera GmbH) with 355 nm wavelength, 1.6 W average power, 3–5 ps pulse duration, 500 kHz repetition rate, and 18 μm full-width at half-maximum spot size. The USP laser system was combined with accurate scanning optics and a computer numerically controlled stage (GFH GmbH) for carrying the polymer substrates. After USP laser micromachining, the surface of the micromachined polymer parts was rough and of poor optical quality that did not allow for an optical read-out or a laser welding. Therefore, the surface of the micromachined polycarbonate substrates was subsequently polished by a wet-chemical treatment (see ref. ^49^ for further information).

After wet-chemical surface polishing, the polycarbonate parts were joined with a blackened thermoplastic polyurethane membrane using laser welding (see Fig. 6c). Alternatively, a pressure-sensitive adhesive foil (Applied Biosystems) was micromachined and subsequently used for the sealing of the micromachined polymer substrate (see Fig. 6b). Both, the adhesive foil and the polyurethane membrane were cutted by a continous wave infrared laser light source, that is a CO_2_ laser (Spirit GLS, GCC LaserPro) with 10.6 μm wavelength and 60 W power. More detailed information regarding the fabrication techniques used for the laser-welded test samples (see Fig. 6c) is provided in ref. ^49^.

After the joining of the polymer parts, the microcavity array chip was ultimately implemented via adhesive bonding (Katiobond 45952, Delo Industrie Klebstoffe) using a hand dispenser (Ultra 2400 Series, Nordson EFD) or a dispensing robot (I&J7300C, Fisnar Inc.). The crosslinking reaction of the adhesive was initiated by an at least 40 s exposure with an UV lamp (US460 lightpen, Unnasol) or a crosslinker (CL-1000 ultraviolet crosslinker, UVP).

### Test setup for rapid thermal cycling and real-time fluorescence read-out of the microfluidic chips

In order to perform PCR experiments within the microfluidic chips, we created a test setup for simultaneous rapid thermal cycling and real-time fluorescence read-out (see Fig. 6d). The setup is based on a fluorescence microscope (BX 61, Olympus K.K.) with a 2.5× magnifying objective for an imaging of the entire microcavity array. On top of the microscope stage, we mounted a homemade apparatus for rapid thermal cycling of the test samples. The apparatus is made up of an aluminum base plate serving as a heat sink, a Peltier device (RS components 693-5107), and a 3d-printed test sample holder. The PID-controlled (Platinum series, OMEGA Engineering Inc.) Peltier device was appropriately integrated into the apparatus to achieve a high heat exchange between the aluminum base plate and the microcavity array chip inside the test sample. During usage, the whole setup was tilted by an angle of 15° against the horizontal. In this way, the buoyancy force was employed to pull disturbing gas bubbles away from the microcavities. Further details regarding the test setup are given in the Supplementary Information.

### Experimental

For the qPCR experiments, 50 μl of a liquid master mix (Illustra Hot Start Mix RTG, GE Healthcare) were slowly introduced into the flow chamber by manual pipetting. The target-specific primers and TaqMan oligonucleotide probes (Biomers GmbH) and template DNA were either introduced with the master mix or prestored inside the microcavities. More detailed information regarding the biocontent and its diagnostic application to chronic myeloid leukemia (CML) is given in ref. ^48^. After sample loading, 150 μl of a fluorinated oil (Fluorinert FC-70, 3M) were pipetted into the flow cell, in order to remove the PCR master mix from the headspace of the flow chamber and to seal the filled microcavities. In this way, an evaporation of the liquid aliquots inside the microcavities could be impeded during the PCR process. Then, the inlet of the flow cell was sealed fluidically tightly with an adhesive tape (VHB GPH-060, 3M). Finally, the loaded microfluidic chip was introduced into the sample holder of the test setup and thermally cycled in order to conduct individual PCRs inside the microcavities. The fluorescence signal generated by the oligonucleotide probes was constantly measured by a charge-coupled device chip using the microscope optics. During the measurements, a fluorescence micrograph (see Fig. 7a for example) was acquired every 10 s each. When using fluorescence probes that are sensitive to photobleaching, a temporally pulsed excitation and fluorescence detection might be preferable.

### Data analysis

For an analysis of the individual qPCRs, the mean gray values of all 96 microcavities were extracted from the raw data using ImageJ (Wayne Rasband, National Institutes of Health). Subsequent to a drift and tilt correction, we applied a customized macro to the entire stack of fluorescence micrographs. As a result, the macro created a .txt file comprising the mean gray values of 96 regions of interest corresponding to the locations of the 96 microcavities. Figure 7a shows a typical fluorescence micrograph (in a contrast adjusted grayscale representation) that was acquired during a qPCR experiment. Figure 7b shows a plot of the extracted fluorescence signal raw data (black curve) that corresponds to the microcavity labeled “F3” in Fig. 7a, which is marked by the white circle.

After data extraction, the individual fluorescence signal curves were further analyzed using Wolfram Mathematica (Wolfram Research). For a deduction of the PCR cycle numbers, we make use of the characteristic temperature dependency of the used TaqMan probe fluorescence signal: we found that the quenching efficiency of the used TaqMan probes decreases with increasing temperature corresponding to an increase of the fluorescence signal when the temperature was raised. In this way, we were able to precisely derive the cycle number directly from the raw data (see gray curve in Fig. 7b).

Next, we calculated the lower envelope (blue curve in Fig. 7b) from each raw data curve, corresponding to the fluorescence at the annealing temperature (60 °C). Then, the lower envelope curve was fitted (red curve in Fig. 7b) by a four-parametric sigmoidal fitting model as described in ref. ^50^:

8 $$F(c)=\frac{\Delta F}{1+\exp (-(c-{c}_{i})/k)}+{F}_{b}$$

where *F* is the actual fluorescence, *c* the actual cycle value, *c*_*i*_ the cycle value corresponding to the inflection point of the curve, *k* a constant describing the width of the fluorescence increase, Δ*F* the total increase in the fluorescence signal *F*, and *F*_*b*_ the background fluorescence signal.

The cycle value *c*_*i*_ that corresponds to the inflection point *F*(*c*_*i*_) of the curve, i.e., ${\partial }^{2}F/\partial {c}^{2}{| }_{c = {c}_{i}}=0$, can be related to the threshold cycle value *c*_*t*_: if the threshold cycle value *c*_*t*_ is defined by a maximum of the curvature, i.e., ${\partial }^{3}F/\partial {c}^{3}{| }_{c = {c}_{t}}=0\ \ \wedge \ \ {\partial }^{4}F/\partial {c}^{4}{| }_{c = {c}_{t}}\,<\,0$, one obtains the relation ${c}_{t}={c}_{i}+k{\mathrm{log}}\,[2-\sqrt{3}]\approx {c}_{i}-1.317\ k$.

Figure 7b depicts a typical raw data curve (shown in black) with a sigmoidal fit (shown in red) of the lower envelope (shown in blue). Based on the fitting parameter sets (*c*_*i*_, *k*, Δ*F*,  *F*_*b*_) of the individual signal curves further data analysis of the PCR array measurement was performed: Fig. 7c shows the normalized sigmoidal fits corresponding to all amplification curves of the PCR array. Figure 7d, e depict a histogram of the *c*_*i*_ values and a map of the spatial distribution of the *c*_*i*_ values, respectively.

## Supplementary information

Supplementary information
