# Supplementary material for: Fusing MEMS technology with lab-on-chip: nanoliter-scale silicon microcavity arrays for digital DNA quantification and multiplex testing
Source: Microsyst Nanoeng. 2020 Oct 5;6:82. doi: 10.1038/s41378-020-00187-1 (PMC8433415; doi:10.1038/s41378-020-00187-1)
Supplement: Supplementary file 1 — Supplementary information [file 41378_2020_187_MOESM1_ESM.pdf]

Supplementary information

for

Fusing MEMS technology with lab-on-chip: nanoliter-scale silicon microcavity arrays for digital DNA quantification and multiplex testing

Daniel Podbiel, Franz Laermer, Jochen Hoffmann

Robert Bosch GmbH, Corporate Sector Research, Microsystems and Nanotechnologies, Robert-Bosch-Campus 1, 71272, Renningen, Germany.
E-mail: DanielSebastian.Podbiel@de.bosch.com

Roland Zengerle

University of Freiburg, IMTEK - Department of Microsystems Engineering, Georges-Koehler-Allee 103, 79110, Freiburg, Germany

1 Supplementary information

1.1 Experimental setup

Fig. S1 (a) shows a photograph of the experimental setup that was used in this study. A commercial fluorescence microscope (BX61, Olympus K.K.) was combined with a home-made compact thermal cycling device that was mounted onto the stage of the microscope. During the experiments, the whole setup was tilted by an angle of 15° against the horizontal by placing a wedge below the microscope.
Fig. S1 (b) sketches the electronic circuit that was used for a precise temperature management of the microfluidic chip. A Peltier device (RS components 693-5107) was controlled by a PID controller (Platinum series, OMEGA Engineering Inc.) using two coil relays (Schrack PT270R24 with relay base Schrack PT78722) for a rapid switching between heating and cooling. The PID controller was connected with a thermocouple (fine gauge exposed welded tip thermocouple - type T (IEC), Z0-PFA-T-1 X 5, Labfacility; Farnell 859-8258) that measured the temperature at the top side of the Peltier device. On top of the Peltier device a thin copper sheet was placed that served as a thermal mass and a low-pass filter for small temperature oscillations caused by the abrupt switching between heating and cooling. In this way, both a rapid thermal cycling and a precise temperature control with minor deviations $< 1°$C were achieved.
The panels (c) and (d) of Fig. S1 show two CAD-based visualizations of the home-made thermal cycling device that was mounted onto the microscope stage. The device was made up of an aluminum base plate serving as a heat sink and a 3d-printed polycarbonate part (shown in blue in panel (c) of Fig. S1), that was mounted onto the base plate via four screws. The 3d-printed part was used both as a sample holder for the microfluidic chips and as a housing for the Peltier device which was located between the base plate and the microfluidic chip. Fig. S1 (d) shows a close-up view of the optical window inside the 3d-printed part. The flow cell and the implemented microcavity array chip are visible as well.
Fig. S1 (e) shows a fluorescence micrograph of a microcavity array chip after filling and sealing the microcavities. The spatial resolution of the setup was appropriately chosen to prevent a possible interference of the optical signals generated by adjacent microcavities. The fluorescence signal from the microcavities is clearly distinguishable from the background signal obtained from the areas between the microcavities. Apart from that, the fluorescence signal shows a slight variation across the field of view, which may be explained by a slightly inhomogeneous illumination. However, the results obtained from the individual quantitative PCR reactions (like the derivated c_i cycles) should not be significantly affected by this small inhomogenity.
Panel (f) of Fig. S1 shows a plot of three temperature curves obtained during thermal cycling of a microfluidic chip inside the test setup. The temperature curves were measured by three thermocouples (fine gauge exposed welded tip thermocouples - type T (IEC), Z0-PFA-T-1 X 5, Labfacility; Farnell 859-8258) located at the top sides of the microcavity array silicon chip (blue curve), the microfluidic chip/test slide (purple curve), and the aluminum base plate (pink curve), respectively. The temperature of the microcavity array silicon chip varied periodically between 95°C (denaturation temperature), 60°C (annealing temperature), and 65°C (elongation temperature) in order to conduct PCRs within the microcavities. In contrast, the temperature of the polymeric test slide did not exceed 75°C because of its low heat conductivity. The aluminum base plate remained nearly constant at some 60°C due to its high thermal mass. Before each PCR experiment, the setup was warmed up to a balanced state in order to ensure comparable and well defined conditions of the setup during the whole thermal cycling.

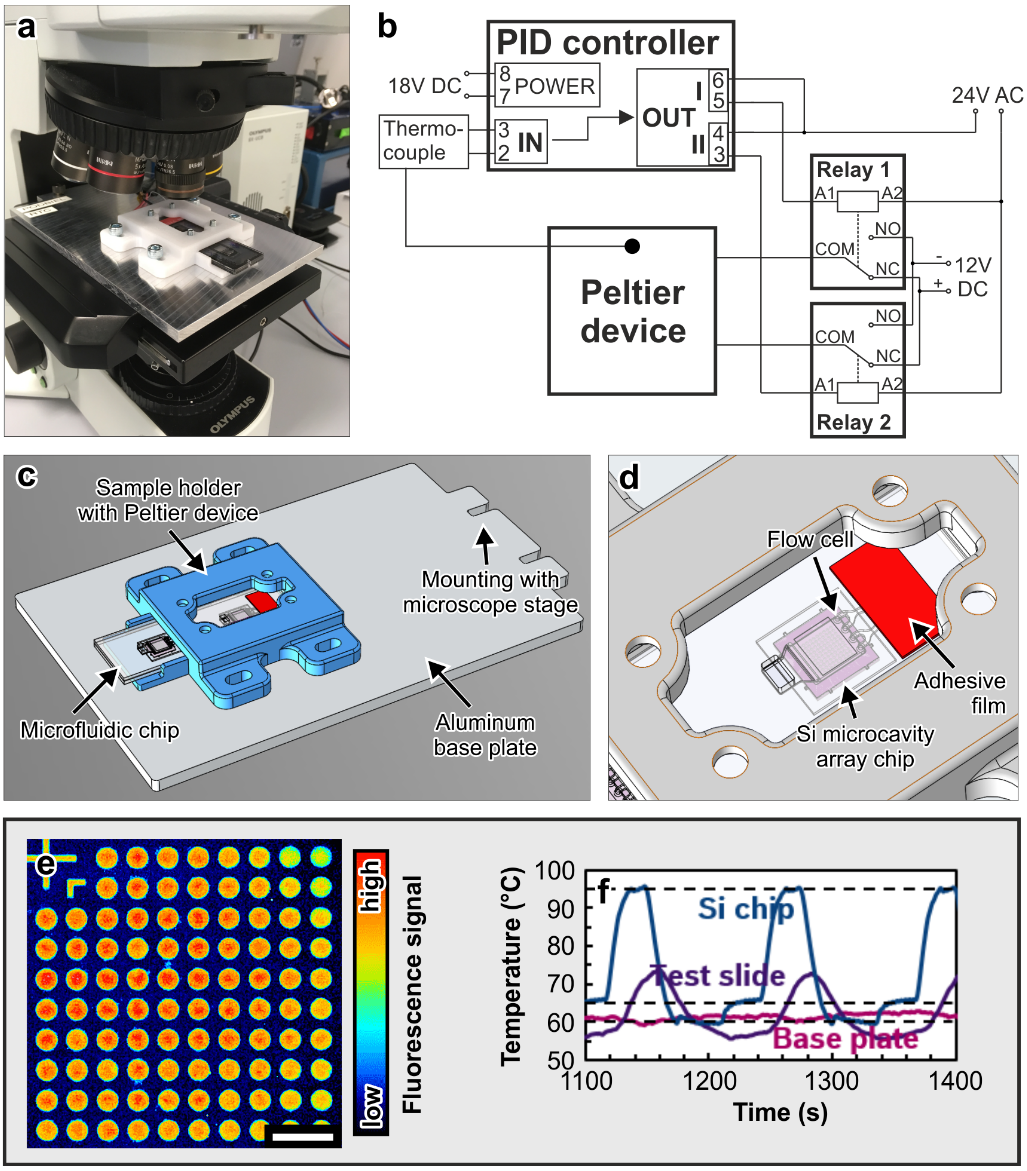

Figure S 1: Experimental setup for simultaneous rapid thermal cycling and real-time optical fluorescence read-out of the microcavity array chips as used for the PCR experiments reported in this work. **a** Photograph of the experimental setup showing the florescence microscope combined with the home-made compact thermal cyling device that is mounted onto the stage of the microscope. **b** Sketch of the control electronics of the Peltier device. **c**: Computer-aided design based visualization of the thermal cycling device. **d**: Close-up view of the optical window for fluorescence read-out of the microcavity array chip. **e**: Fluorescence micrograph of a test sample after filling and sealing of the microcavity array. The scalebar corresponds to 1 mm. **f**: Temperatures at the silicon chip (blue curve), the test slide (pruple curve), and the base plate (pink curve) during thermal cycling as measured by thermocouples.

1.2 Description of the amplification statistics by continous copy numbers and matrix formalism

The discrete description using sums can be replaced by a continous one using integral terms if the binomial coefficient in Eq. (1) is expressed for non-integer values of c by making use of the Beta function

$$B(x,y) = \int_0^1 dt\, t^{x-1}\,(1-t)^{y-1}$$

in the form

$$\binom{n}{k} = [(n+1)B(n-k+1,k+1)]^{-1}$$

In this way, non-integer values of $\bar{c}$ and c_{LOD} are included. The extension is straightforward by replacing the discrete sums over the copy number by continous integral terms. Hence, Eqs. (2), (4), and (7) become

$$r(\bar{c}) = \int_0^\infty dc\, p_d(c)\cdot B_n(c,\bar{c})$$

$$r_{\Theta;\,\mathrm{LOD}}(\bar{c}) = \int_{c_{\mathrm{LOD}}}^\infty dc\, B_n(c,\bar{c})$$

and

$$r_{G;\,\mathrm{LOD},w}(\bar{c}) = \int_0^\infty dc \int_{-\infty}^{c-c_{\mathrm{LOD}}} dc'\, G_{w,0}(c')\cdot B_n(c,\bar{c})$$

respectively. Apart from that, a matrix formalism can be used where the positive rate is described by a vector

$$r_{\bar{c}} = \sum_c B^n_{\bar{c}c}\cdot p^d_c$$

where $B^n_{\bar{c}c}$ is the binomial probability density distribution matrix and p^d_c the probability of detection vector. By making use of the inverse binomial probability density distribution matrix $(B^n_{\bar{c}c})^{-1}$ an experimental POD vector $p^{d,e}_c$ can be calculated from the experimentally determined positive rates r^e via

$$p^{d,e}_c = \sum_{\bar{c}} (B^n_{\bar{c}c})^{-1}\cdot r_{\bar{c}}$$

1.3 Determination of reagent carry-over during microfluidic filling and sealing by the prestorage of template DNA in selected microcavities

Besides the chessboard pattern presented in section 2.4 of the main text, we also investigated a second layout where some 100 copies of ABL template DNA were prestored in four selected microcavities of the array each as sketched in Fig. S2 (a). Again, the microcavities with prestored template DNA inside exhibited a higher fluorescence level during thermal cycling indicating a PCR amplification as highlighted by the orange circles in panel (b) of Fig. S2. Fig. S2 (c) reveals that false positives are delayed by at least 8 PCR cycles. Hence, the carry-over of prestored template DNA is very low here, which is consistent with the previous results given in section 2.4.

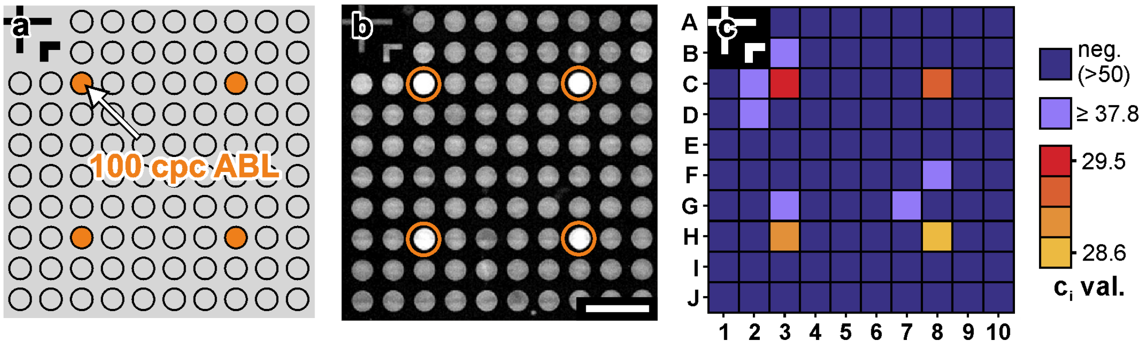

Figure S 2: Experimental investigation of reagent carry-over during filling and sealing by the PCR-based amplification of prestored ABL template DNA within the microcavity array. **a** Spotting layout. Four selected microcavities contain some 100 copies of ABL template DNA. **b** Fluorescence micrograph acquired during thermal cycling at annealing temperature (60°C). The scalebar corresponds to 1 mm. **c**: False-color map of the c_i values of the polymerase chain reactions inside the individual microcavities. The microcavities where no PCR amplification could be detected within 50 temperature cycles are indicated in dark blue.
